# A radiomics signature derived from CT imaging to predict MSI status and immunotherapy outcomes in gastric cancer: a multi-cohort study

**DOI:** 10.1186/s12885-024-12174-0

**Published:** 2024-04-01

**Authors:** Peng-chao Zhan, Shuo Yang, Xing Liu, Yu-yuan Zhang, Rui Wang, Jia-xing Wang, Qing-ya Qiu, Yu Gao, Dong-bo Lv, Li-ming Li, Cheng-long Luo, Zhi-wei Hu, Zhen Li, Pei-jie Lyu, Pan Liang, Jian-bo Gao

**Affiliations:** 1 Department of Radiology, The First Affiliated Hospital of Zhengzhou University, No.1 Jianshe Road, 450052 Zhengzhou, Henan PR China; 2 Department of Radiology, The Second Hospital, Cheello College of Medicine, Shandong University, 250033 Jinan, PR China; 3 Department of Interventional Radiology, The First Affiliated Hospital of Zhengzhou University, 450052 Zhengzhou, Henan PR China; 4 Department of Interventional Medicine, The Second Hospital, Cheello College of Medicine, Shandong University, 250033 Jinan, Shandong PR China; 5 Zhengzhou University Medical College, 450052 Zhengzhou, Henan PR China

**Keywords:** Gastric cancer, MSI, Immunotherapy, Radiomics signature, mRNA-seq

## Abstract

**Background:**

Accurate microsatellite instability (MSI) testing is essential for identifying gastric cancer (GC) patients eligible for immunotherapy. We aimed to develop and validate a CT-based radiomics signature to predict MSI and immunotherapy outcomes in GC.

**Methods:**

This retrospective multicohort study included a total of 457 GC patients from two independent medical centers in China and The Cancer Imaging Archive (TCIA) databases. The primary cohort (*n* = 201, center 1, 2017–2022), was used for signature development via Least Absolute Shrinkage and Selection Operator (LASSO) and logistic regression analysis. Two independent immunotherapy cohorts, one from center 1 (*n* = 184, 2018–2021) and another from center 2 (*n* = 43, 2020–2021), were utilized to assess the signature’s association with immunotherapy response and survival. Diagnostic efficiency was evaluated using the area under the receiver operating characteristic curve (AUC), and survival outcomes were analyzed via the Kaplan-Meier method. The TCIA cohort (*n* = 29) was included to evaluate the immune infiltration landscape of the radiomics signature subgroups using both CT images and mRNA sequencing data.

**Results:**

Nine radiomics features were identified for signature development, exhibiting excellent discriminative performance in both the training (AUC: 0.851, 95%CI: 0.782, 0.919) and validation cohorts (AUC: 0.816, 95%CI: 0.706, 0.926). The radscore, calculated using the signature, demonstrated strong predictive abilities for objective response in immunotherapy cohorts (AUC: 0.734, 95%CI: 0.662, 0.806; AUC: 0.724, 95%CI: 0.572, 0.877). Additionally, the radscore showed a significant association with PFS and OS, with GC patients with a low radscore experiencing a significant survival benefit from immunotherapy. Immune infiltration analysis revealed significantly higher levels of CD8 + T cells, activated CD4 + B cells, and TNFRSF18 expression in the low radscore group, while the high radscore group exhibited higher levels of T cells regulatory and HHLA2 expression.

**Conclusion:**

This study developed a robust radiomics signature with the potential to serve as a non-invasive biomarker for GC’s MSI status and immunotherapy response, demonstrating notable links to post-immunotherapy PFS and OS. Additionally, distinct immune profiles were observed between low and high radscore groups, highlighting their potential clinical implications.

**Supplementary Information:**

The online version contains supplementary material available at 10.1186/s12885-024-12174-0.

## Background

Gastric cancer (GC) ranks as the fifth most prevalent cancer and the fourth leading cause of cancer-related death globally, contributing to approximately 770,000 annual fatalities [1]. While surgical resection followed by adjuvant chemotherapy is established as an effective treatment for early-stage GC, a significant proportion of patients are diagnosed beyond this stage due to the disease’s insidious onset and rapid progression [2, 3]. Palliative chemotherapy serves as the mainstay treatment for unresectable or metastatic GC patients, yet its clinical benefits are very limited, resulting in a median survival time of less than one year [4, 5].

In recent years, tumor immunotherapy has made remarkable strides in both research and clinical practice, offering a promising treatment avenue for patients with unresectable or metastatic gastric cancer [4, 6, 7]. The combination of PD-1/PD-L1 inhibitors and chemotherapy has now become the standard first-line treatment for GC in major guidelines [8–10]. Unfortunately, due to the strong heterogeneity of gastric cancer, only a minority of GC patients respond to current immunotherapy treatment [11]. Thus, there is an urgent need to identify reliable biomarkers for screening GC patients likely to benefit from immunotherapy.

Microsatellite instability (MSI) stands out as the first pan-cancer immune biomarker approved by the Food and Drug Administration (FDA), with solid tumors exhibiting MSI-high (MSI-H) recommended for immunotherapy [12, 13]. The MSI-H phenotype generates numerous immunogenic neoantigens detected by the immune system, rendering MSI status a valuable clinical biomarker for checkpoint immunotherapy [14]. Presently, MSI status assessment primarily relies on immunohistochemistry or polymerase chain reaction (PCR) analysis of specimens obtained through endoscopic biopsy or surgical resection [15]. However, information on MSI expression status obtained postoperatively has limited impact on treatment planning prior to surgery. Moreover, the limited samples obtained through biopsy may not comprehensively reflect tumor heterogeneity, leading to false-negative results (2.1–5.9%) [16, 17]. Additionally, biopsies and surgeries are invasive, time-consuming, expensive, and carry risks of complications, making repeated monitoring inconvenient [17]. Hence, there is an urgent need to develop a non-invasive, reliable, and cost-effective method to identify MSI status.

Radiomics is a rapidly advancing field that utilizes advanced computational techniques to transform medical images, such as CT and MRI, into quantitative features, enabling the development of a signature for cancer diagnosis and treatment [18, 19]. While several studies have demonstrated the potential and significance of radiomics in evaluating the MSI status of gastric cancer, these investigations have not thoroughly delved into the clinical value of their radiomics models within cohorts of patients undergoing immunotherapy [20–22]. Specifically, there is a lack of comprehensive validation regarding the efficacy of radiomics models in predicting immunotherapy outcomes. Moreover, the aforementioned studies did not delve into the potential biological value of radiomics models from the perspectives of the immune microenvironment and transcriptome.

Therefore, this study aims to fill the gaps in the current research landscape by establishing a non-invasive radiomics biomarker. This biomarker will not only identify MSI status, but will also undergo further validation in cohorts of patients undergoing immunotherapy, which will offer a comprehensive understanding of its practical feasibility. Moreover, we will analyze the immune microenvironment features of patients stratified into different radscore groups based on transcriptomic data, providing a deeper understanding of the predictive mechanisms of the radiomics model.

## Materials and methods

### Patients

The study received approval from the local Ethics Committee (2022-KY-1447-002) in accordance with the Declaration of Helsinki. Written informed consent was waived by the Human Scientific Ethics Committee of the First Affiliated Hospital of Zhengzhou University due to the retrospective design of the study.

**Fig. 1:**
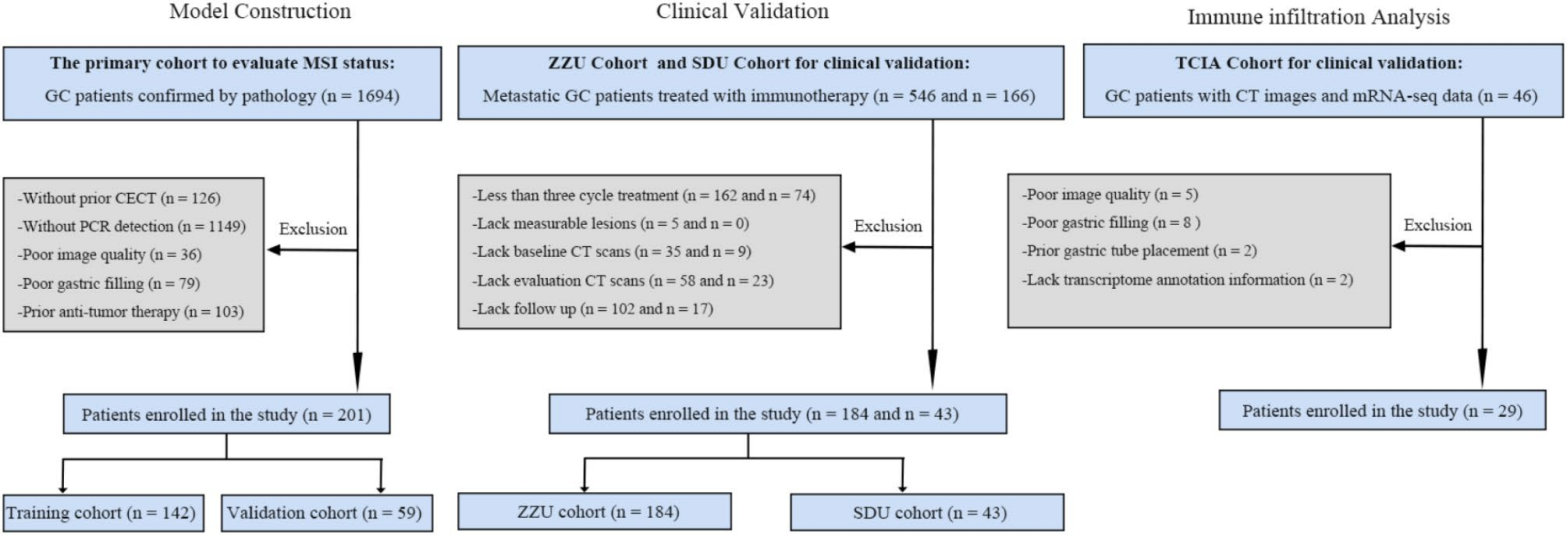
Patient flowchart for this study. MSI = microsatellite instability, GC = gastric cancer, PCR = polymerase chain reaction

Patient selection process is depicted in Fig. 1. To develop the radiomics signature associated with MSI status, the study retrospectively enrolled 201 consecutive gastric cancer (GC) patients from Center 1 (the First Affiliated Hospital of Zhengzhou University, 2017–2022). MSI status was determined using PCR detection. The primary cohort was randomly divided into a training cohort (*n* = 142) and a validation cohort (*n* = 59) at a 7:3 ratio. In addition, two independent immunotherapy cohorts were employed to investigate the association of the radiomics signature with immunotherapy response and validate its prognostic value. The first cohort, ZZU cohort, comprised 184 GC patients treated at Center 1 between 2018 and 2021. The second cohort, SDU cohort, included 43 GC patients treated between 2020 and 2021 at Center 2 (the Second Hospital of Shandong University). Furthermore, one GC cohort (*n* = 29) from The Cancer Genome Atlas (TCGA) and The Cancer Imaging Archive (TCIA) databases was included in the study to further evaluate the immune infiltration landscape across different subgroups of the radiomics signature. Supplementary A outlines the inclusion and exclusion criteria for all cohorts, and Supplementary B provides information on the immunotherapy regimens used in the immunotherapy cohorts.

### Detection of MSI status

MSI status was determined by performing PCR testing on tumor tissue obtained from surgical resection or biopsy. Five Bethesda microsatellite markers (BAT25, BAT26, D2S123, D5S346, and D17S250) were utilized to assess MSI status. Tumors with instability at two or more of these five markers were classified as MSI-H, while those without instability or with instability at only one marker were classified as MSS or MSI-L. In this study, we grouped tumors with MSI-L and MSS together as the MSS group due to their similar clinical significance.

### CT acquisition and radiologic evaluation

CT scan parameters are detailed in Supplementary C. A consensus review of all images was conducted by two radiologists, LL and LP, who evaluated the clinical T stage and clinical N stage of each patient based on the AJCC version 8 TNM staging system. LL had 5 years of experience in abdominal CT imaging, while LP had 10 years of experience. Any discrepancies in radiologic evaluation were resolved through discussion and consultation with a third senior radiologist, GJ, who had over 20 years of experience in abdominal CT imaging. The radiologists were blinded to clinical information during the evaluation process.

### ROI segmentation and feature extraction

Portal venous phase CT images of all patients were analyzed using the open-source software 3D Slicer (version 4.13.0; https://www.slicer.org/). Manual ROI segmentations were performed by outlining the tumor lesion’s margin on the largest slice. To ensure intra-observer reliability, ZP performed the ROI segmentation and feature extraction process twice within a week for CT images of 50 randomly selected patients. For inter-observer reliability assessment, two radiologists, ZP and YS, independently conducted ROI segmentation and feature extraction on medical images of the same 50 patients. ZP completed the remaining ROI segmentation independently.

To minimize the influence of different scanning schemes or equipment on quantitative radiomics features, the ROI segmented images were resampled to a voxel size of 1 × 1 × 1 mm^3^ and discretized using a bin width of 25 for gray level discretization [19]. A total of 851 radiomics features were extracted from each ROI segmented image. Following feature extraction, all radiomics features underwent standardized using the z-score for subsequent analysis.

### Feature selection, model construction and evaluation

All 851 radiomics features were analyzed further based on the training cohort. To reduce the dimensionality of radiomics features and identify robust predictors of MSI status, we utilized a meticulous three-step procedure. Firstly, we calculated the inter-observer and intra-observer correlation coefficients (ICCs) for each feature and retained those with ICCs greater than 0.8 for further analysis. Secondly, a multivariate ranking method called maximum relevance minimum redundancy (mRMR) algorithm was applied to eliminate redundant and irrelevant features on the basis of a heuristic scoring criterion, and only the top ranked 200 features were retained. Lastly, the least absolute shrinkage and selection operator (LASSO) logistic regression algorithm was performed to choose the most valuable subset of features from the top ranked 200 features. The regular parameter (λ) of LASSO regression was chosen when the average mean square error was minimal by 10-fold cross-validation. Moreover, the most valuable subset of features was utilized to create the radiomics signature in the training cohort.

Clinical risk factors associated with MSI status were identified through univariate analysis with a significance threshold of *p* < 0.05. Based on the clinical risk factors and radiomics signature, we developed three models through multivariable logistic regression: the clinical model, the radiomics model, and the combined model. Discrimination performance was assessed by calculating the area under the receiver operating characteristic curve (AUC), and the results were compared using the non-parametric Delong test. The predictive accuracy of the radiomics signature was further evaluated in both the training and validation cohorts using calibration curves and the Hosmer-Lemeshow test. Furthermore, a nested 5-fold cross-validation approach was conducted on the selected radiomics features using logistic regression to further assess the performance of the radiomics signature. In the outer 5-fold cross-validation loop, we divided the data into training (4-folds) and test (1-fold) datasets to evaluate the performance of the models with an untouched test set. In each training step of the outer fold, an inner 10-fold cross-validation was applied using all the selected radiomics features to tune the hyperparameters and select the optimal logistic regression model. The nested 5-fold cross-validation process was repeated 5 times to ensure the robustness of our evaluation results.

### Association between radiomics signature and immunotherapy outcomes

Patients’ immunotherapy responses were categorized into four groups according to the RECIST 1.1 guidelines: complete response (CR), partial response (PR), stable disease (SD), or progressive disease (PD). The immunotherapy cohort patients were then grouped into two categories based on their treatment response: the CR/PR group and the PD/SD group. The radiomics signature calculated a radscore for each patient in the immunotherapy cohorts, and its ability to predict a CR or PR response to immunotherapy was evaluated using AUC value. Additionally, the prognostic significance of the radiomics signature was evaluated through Kaplan-Meier survival curves and log-rank tests. Progression-free survival (PFS) was defined as the time from the initiation of immunotherapy to disease progression, while overall survival (OS) was defined as the duration from the initiation of immunotherapy to disease-related death or the last follow-up date.

### Immune infiltration assessment

In the TCIA cohort, patients were categorized into a high radscore group (≥ the median) and a low radscore group (< the median). Utilizing mRNA sequencing data, the CIBERSORT algorithm estimated the abundance of 22 immune cell in the tumor immune micro-environment (TME). Furthermore, we assessed differences between the two groups concerning MSI status and immune-regulating factors, including co-stimulators, co-inhibitors, and other relevant factors. Immune regulators are known to play a crucial role in modulating the function of immune cells, exerting either anti-tumor or pro-tumor effects.

### Statistical analysis

Statistical analyses were conducted using R software (version 4.2.2, https://www.r-project.org/). Continuous variables, if normally distributed, were reported as mean  ±  standard deviation; otherwise, they were expressed as median (lower quartile, upper quartile). Fisher’s exact tests were applied to categorical variables, while appropriate tests such as Student’s t-test or Mann-Whitney U test were used for continuous variables. ICC analysis, mRMR algorithm, and LASSO regression analysis were conducted using the “irr”, “mRMRe”, and “glmnet” packages, respectively. Receiver operating characteristic (ROC) curves were generated using the “pROC” and “ggplot2” packages. The nested 5-fold cross-validation was performed by applying “nestedcv” package. The prognostic value of the radiomics signature was assessed using Kaplan-Meier survival curves and log-rank tests. The “survminer” package was used to plot the survival curves, and the “IOBR” package [23] was conducted for immune infiltration analysis. The MSI status in the TCIA cohort was obtained using the “cBioPortalData” package, and a threshold of 0.4 was applied to distinguish between MSI and MSS [24, 25]. Statistical significance was defined as a *p*-value of less than 0.05.

## Results

### Patient characteristics

A total of 457 GC patients were enrolled in this study. Demographic and clinical characteristics of the training cohort (*n* = 142) and validation cohort (*n* = 59) are summarized in Table 1. Within the patient population, 149 were male, 77 were aged 65 years or older, and the MSI-H positivity rate was 22.4%.

**Table 1:** Characteristics of patients used to evaluate MSI status

	Training cohort (*n* = 142)			Validation cohort (*n* = 59)		
Characteristic	MSI-H	MSS	*p* value	MSI-H	MSS	*p* value
**Age (years)**			1			0.738
< 65	19 (59.37)	65 (59.09)		8 (61.54)	32 (69.57)	
≥ 65	13 (40.63)	45 (40.91)		5 (38.46)	14 (30.43)	
**Sex**			0.644			0.037
Male	23 (71.88)	84 (76.36)		6 (46.15)	36 (78.26)	
Female	9 (28.12)	26 (23.64)		7 (53.85)	10 (21.74)	
**Location**			0.007			0.015
Cardia	5 (15.63)	40 (36.36)		1 (7.69)	18 (39.13)	
Body	6 (18.75)	34 (30.91)		4 (30.77)	19 (41.31)	
Antrum	20 (62.50)	32 (29.09)		7 (53.85)	8 (17.39)	
Whole	1 (3.12)	4 (3.64)		1 (7.69)	1 (2.17)	
**Clinical T stage**			0.013			0.762
T3	13 (40.63)	73 (66.36)		7 (53.85)	27 (58.70)	
T4	19 (59.37)	37 (33.64)		6 (46.15)	19 (41.30)	
**Clinical N stage**			0.062			0.113
N-	17 (53.13)	37 (33.64)		10 (76.92)	22 (47.83)	
N+	15 (46.87)	73 (66.36)		3 (23.08)	24 (52.17)	
**CA 72−4 level**			0.290			0.187
Normal	24 (75.00)	93 (84.55)		7 (53.85)	34 (73.91)	
Abnormal	8 (25.00)	17 (15.45)		6 (46.15)	12 (26.09)	
**CA 19−9 level**			0.605			0.357
Normal	25 (78.13)	91 (82.73)		10 (76.92)	41 (89.13)	
Abnormal	7 (21.87)	19 (17.27)		3 (23.08)	5 (10.87)	
**CEA level**			0.443			1.00
Normal	25 (78.13)	92 (83.64)		11 (84.62)	40 (86.96)	
Abnormal	7 (21.87)	18 (16.36)		2 (15.38)	6 (13.04)	
Radiomics score *	0.91 (0.50, 1.22)	1.53 (1.22, 1.87)	< 0.001	0.85 (0.43, 1.12)	1.49 (0.94, 1.67)	< 0.001

*Note*: Except where indicated, data are number (%) of patients. MSI-H = microsatellite instability-high, MSS = microsatellite stability, CA 72 − 4 = carbohydrate antigen 72 − 4, CA 19 − 9 = carbohydrate antigen 19 − 9, CEA = carcinoembryonic antigen

* Data in parentheses are interquartile range

Clinical characteristics of the two immunotherapy cohorts are presented in Table 2. In the ZZU cohort, 132 patients were included, with 71.7% being male and 38.0% aged 65 years or older. Among these patients, 64 (34.8%) achieved CR or PR with immunotherapy, with median PFS and OS of 5.8 (2.9, 10.7) months and 10.8 (6.0, 16.2) months, respectively. The SDU cohort comprised 43 patients, with 81.4% being male, and none were aged 65 years or older. Among these patients, 18 (41.9%) achieved CR or PR with immunotherapy, and the median PFS and OS were 5.8 (3.7, 7.9) months and 9.5 (7.2, 14.3) months, respectively.

**Table 2:** Characteristics for immunotherapy cohorts

	ZZU cohort (*n* = 184)			SDU cohort (*n* = 43)		
Characteristic	CR + PR	SD + PD	*p* value	CR + PR	SD + PD	*p* value
**Age (years)**			0.874			> 0.999
< 65	39 (60.94)	75 (62.50)		18 (100)	25 (100)	
≥ 65	25 (39.06)	45 (37.50)		0 (0)	0 (0)	
**Sex**			0.308			0.701
Male	49 (76.56)	83 (69.17)		14 (77.78)	21 (84.00)	
Female	15 (23.44)	37 (30.83)		4 (22.22)	4 (16.00)	
**Location**			0.189			0.624
Cardia	39 (60.94)	54 (45.00)		6 (33.33)	12 (48.00)	
Body	12 (18.75)	33 (27.50)		5 (27.78)	7 (28.00)	
Antrum	13 (20.31)	31 (25.83)		6 (33.33)	4 (16.00)	
Whole	0 (0.00)	2 (1.67)		1 (5.6)	2 (8.00)	
**Clinical T stage**			0.366			> 0.999
T3	18 (28.12)	26 (21.67)		0 (0)	0 (0)	
T4	46 (71.88)	94 (78.33)		18 (100)	25 (100)	
**Clinical N stage**			0.457			0.253
N-	16 (25.00)	24 (20.00)		0 (0)	3 (12.00)	
N+	48 (75.00)	96 (80.00)		18 (100)	22 (88.00)	
**CA 72−4 level**			0.872			> 0.999
Normal	21 (32.81)	41 (34.17)		4 (22.22)	5 (20.00)	
Abnormal	43 (67.19)	79 (65.83)		14 (77.78)	20 (80.00)	
**CA 19−9 level**			0.498			0.765
Normal	16 (25.00)	36 (30.00)		7 (38.89)	11 (44.00)	
Abnormal	48 (75.00)	84 (70.00)		11 (61.11)	14 (56.00)	
**CEA level**			0.640			0.099
Normal	28 (43.75)	48 (40.00)		15 (83.33)	14 (56.00)	
Abnormal	36 (56.25)	72 (60.00)		3 (16.67)	11 (44.00)	
Radiomics score *	1.06 (0.69, 1.35)	1.47 (1.18, 1.87)	< 0.001	1.02 (0.03, 1.34)	1.42 (1.00, 1.93)	0.012

*Note*: Except where indicated, data are number (%) of patients. MSI-H = microsatellite instability-high, MSS = microsatellite stability, CA 72 − 4 = carbohydrate antigen 72 − 4, CA 19 − 9 = carbohydrate antigen 19 − 9, CEA = carcinoembryonic antigen, PFS = Progression-free survival, OS = Overall survival

* Data in parentheses are interquartile range

Baseline data for the TCIA cohort (*n* = 29) is provided in **Supplementary Table**E1. This cohort consisted of 29 patients, with 85.7% being male and 78.6% aged 60 years or older. The median radscore in this cohort was 1.45 (0.87, 1.84), and 14 (48.3%) patients were classified into the low radscore group.

### Development and validation of the radiomics signature

**Fig. 2:**
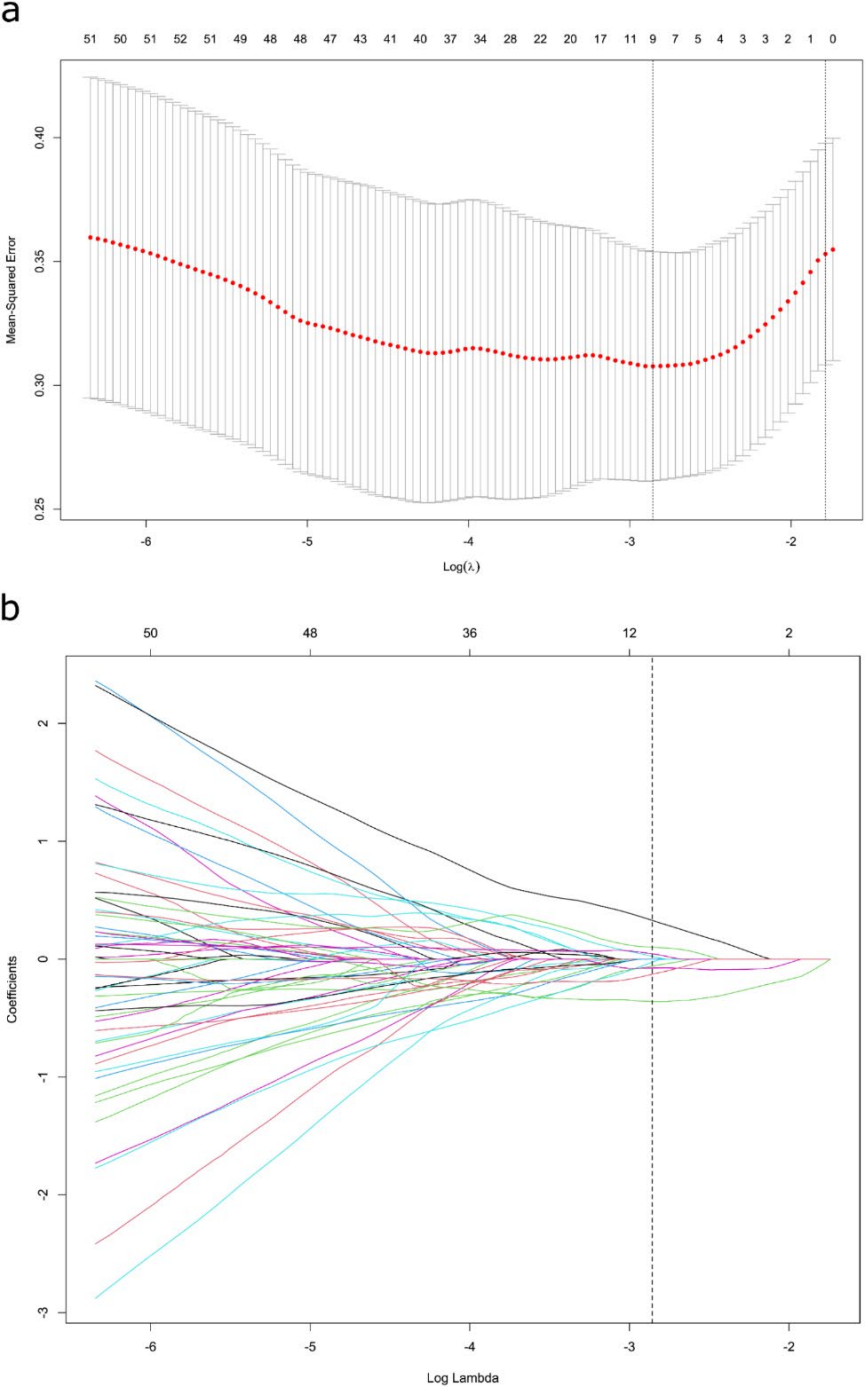
Radiomics feature selection by using the least absolute shrinkage and selection operator (LASSO) logistic regression. (**a**) The selection of tuning parameter (λ) in the LASSO model used 10-fold cross-validation via minimum criteria. The AUC curve was plotted versus log (λ). (**b**) LASSO coefficient profiles of the radiomics features. A vertical line was plotted at the optimal λ value, which resulted in 9 features with nonzero coefficients

Significant associations between tumor location and MSI-H expression were identified in both the training (*p* = 0.007) and validation cohorts (*p* = 0.015) through univariate analysis of clinical factors. To enhance robustness and eliminate redundancy, ICC analysis and the mRMR algorithm were employed to eliminate radiomics features. Subsequently, a 10-fold cross-validation LASSO algorithm (Fig. 2) was applied to develop a radiomics signature, comprising nine features. Supplementary Table E2 provides the coefficients of each feature in the radiomics signature. Clinical and radiomics models were independently created based on tumor location and radiomics signature. The combined model was developed by integrating tumor location and radiomics signature using multivariate logistic regression (Table 3). Detailed explanations for the three logistic regression models are presented in Supplementary Results.

**Table 3:** Multivariable logistic regression analysis of predictors for MSI status

Characteristic (reference level)	Odds Ratio	95% CI	p value
**Location (Cardia)**			
Body	1.52	0.34–7.17	0.581
Antrum	3.60	1.06–14.45	0.051
Whole	0.33	0.01–8.20	0.560
Radiomics signature	0.08	0.02–0.22	< 0.001

*Note*: Data in parentheses are reference level. MSI = microsatellite instability, CI = confidence intervals

**Fig. 3:**
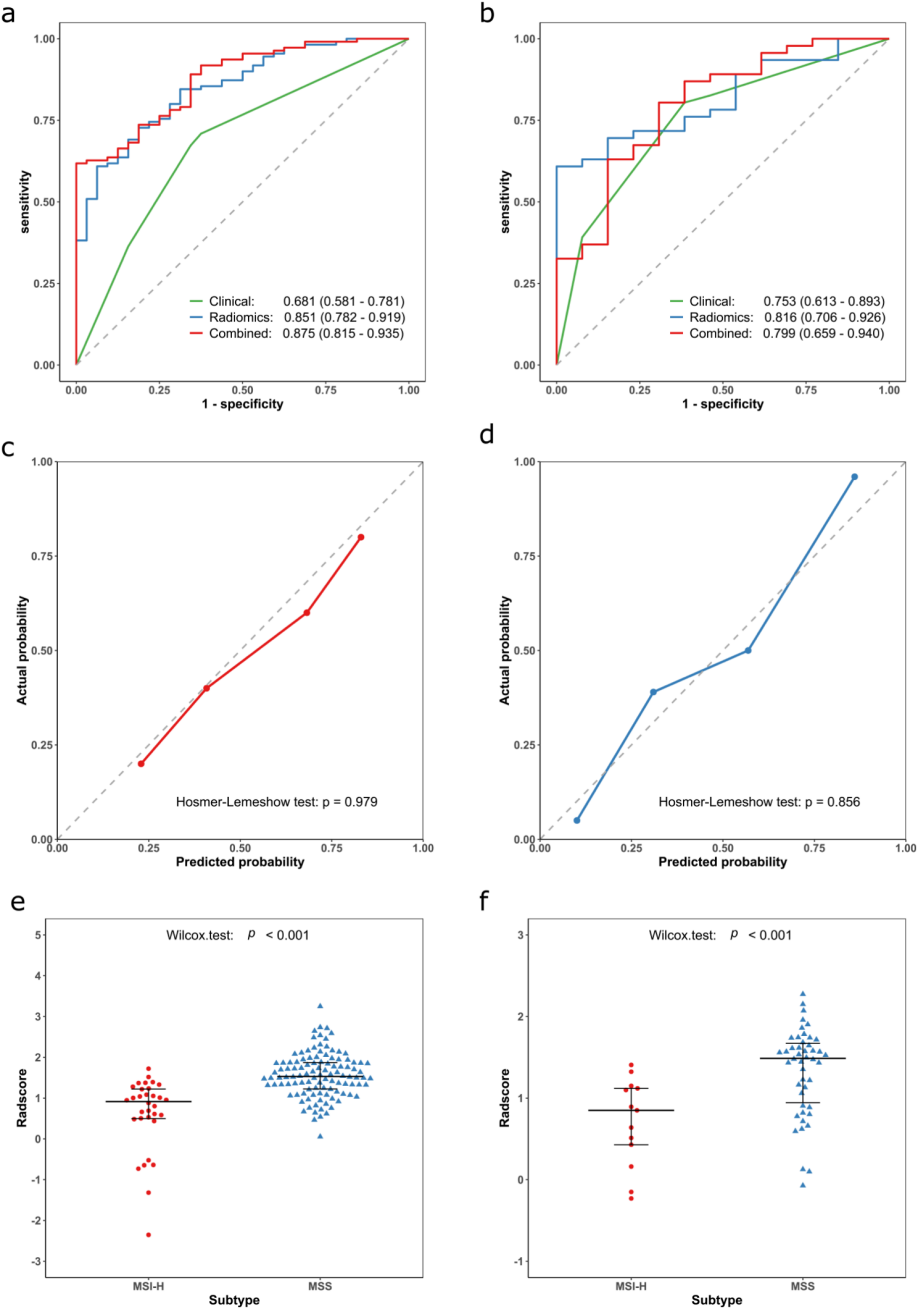
Receiver operating characteristic curves (ROC) for different models in the training (**a**), and validation cohorts (**b**); Calibration curves for the radiomics signature in the training (**c**), and validation cohorts (**d**); Radscore of different subtypes in the training (**e**), and validation cohorts (**f**). MSI-H = microsatellite instability-high, MSS = microsatellite stable

Table 4 presents the diagnostic performance indicators, including accuracy, sensitivity, and specificity of the three models in both the training and validation cohorts. ROC curves for MSI-H positive expression are shown in Fig. 3a and b, and the results of the Delong test are presented in Supplementary Table E3. The analysis demonstrated that the radiomics signature exhibited good predictive ability, with an AUC value of 0.851 (95% CI: 0.782, 0.919) in the training cohort and an AUC value of 0.816 (95% CI: 0.706, 0.926) in the validation cohort. Calibration curves (Fig. 3c and d) demonstrated good consistency between the actual and predicted probabilities of the radiomics signature, and the Hosmer-Lemeshow test indicated a good model fit (*p* > 0.05). The radscore of the MSI-H group calculated by the radiomics signature was significantly lower than that of the MSS group in both the training (median: 0.91 (0.50, 1.22) vs. 1.53 (1.22, 1.87)) and validation cohorts (median: 0.85 (0.43, 1.12) vs. 1.49 (0.94, 1.67)), as shown in Fig. 3e and f (*p* < 0.001). Finally, the results of the nested 5-fold cross-validation (**Supplementary Results**) indicated that the radiomics signature’s performance remained reliable.

**Table 4:** The specific performances of models for evaluate MSI status

Model	Cohort	AUC	Accuracy	Sensitivity	Specificity
Clinical	Training	0.681 (0.581, 0.781)	0.690	0.709	0.625
	Validation	0.753 (0.613, 0.893)	0.763	0.804	0.615
Radiomics signature	Training	0.851 (0.782, 0.919)	0.683	0.938	0.609
	Validation	0.816 (0.706, 0.926)	0.695	1	0.608
Combined	Training	0.875 (0.815, 0.935)	0.704	1	0.618
	Validation	0.799 (0.659, 0.940)	0.780	0.804	0.692

*Note*: Data in parentheses are 95% CI. MSI = microsatellite instability, AUC = area under the receiver operating characteristic curve, CI = confidence intervals

### **Clinical prognostic validation of radiomics signature in immunotherapy**

**Fig. 4:**
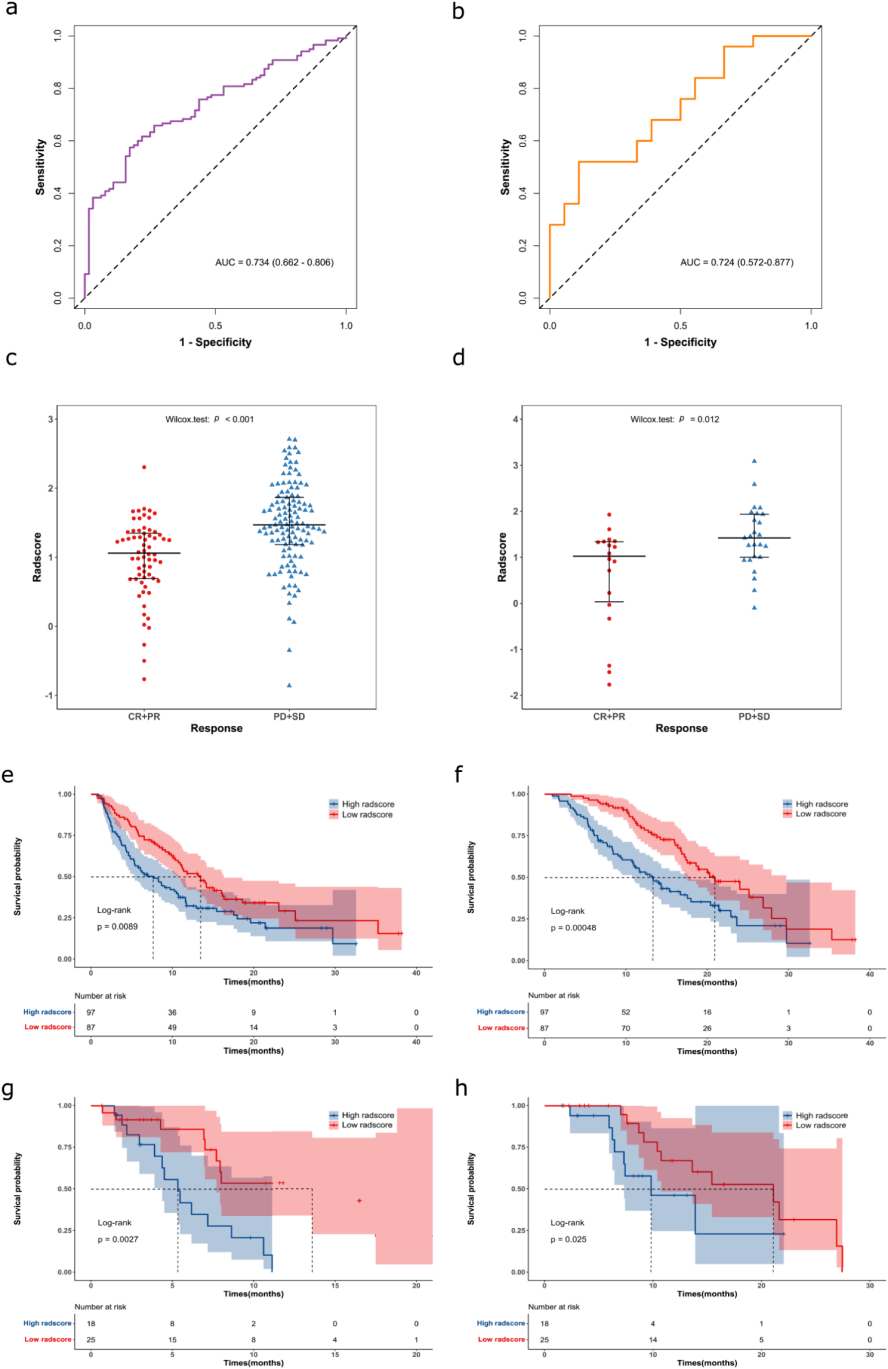
Receiver operating characteristic curves (ROC) illustrating the predictive performance of the radiomics signature for immunotherapy response in the ZZU (**a**) and SDU cohorts (**b**). Radscore distribution for different immunotherapy responses in the ZZU (**c**) and SDU cohorts (**d**). Kaplan-Meier analysis of progression-free survival (PFS) and overall survival (OS) based on distinct radscore groups in the immunotherapy cohorts: (**e**) PFS stratified by radscore groups in the ZZU cohort; (**f**) OS stratified by radscore groups in the ZZU cohort; (**g**) PFS stratified by radscore groups in the SDU cohort; (**h**) OS stratified by radscore groups in the SDU cohort. CR = complete response, PR = partial response, SD = stable disease, PD = progressive disease

The ZZU cohort exhibited a significant lower radscore (median: 1.06 (0.69, 1.35)) in the CR/PR group compared to the PD/SD group (median: 1.47 (1.18, 1.87)) (*p* < 0.001). The AUC value for predicting CR/PR after immunotherapy was 0.734 (95% CI: 0.662, 0.806). Similarly, in the SDU cohort, the CR/PR group demonstrated a significantly lower radscore (median: 1.02 (0.03, 1.34)) compared to the PD/SD group (median: 1.42 (1.00, 1.93)) (*p* = 0.012). The AUC value for predicting CR/PR after immunotherapy in this cohort was 0.724 (95% CI: 0.572, 0.877). The results are presented in Fig. 4a-d.

Furthermore, the results from the ZZU and SDU cohorts revealed that patients in the low radscore group had a significantly longer median PFS of 8.3 months (4.6, 12.8) and 7.4 months (4.9, 8.0) and a longer median OS of 14.0 months (10.4, 18.7) and 12.2 months (9.0, 21.2), respectively, compared to those in the high radscore group. The high radscore group had a median PFS of 4.6 months (2.4, 8.7) and 4.9 months (3.2, 6.9) and a median OS of 7.7 months (5.3, 12.7) and 6.9 months (6.2, 8.0).

The results of Kaplan-Meier analysis revealed a significant association between the radiomics signature and both PFS (HR: 1.59 (95% CI: 1.12, 2.27), *p* = 0.009) and OS (HR: 2.00 (95% CI: 1.34, 2.98), *p* < 0.001) in the ZZU cohort (Fig. 4e and f). Similarly, in the SDU cohort (Fig. 4g and h), the radiomics signature was significantly associated with both PFS (HR: 3.12 (95% CI: 1.31, 7.40), *p* = 0.003) and OS (HR: 2.51 (95% CI: 0.86, 7.35), *p* = 0.025). Importantly, patients in the low radscore group, predicted by the radiomics signature, experienced a significantly longer median PFS and OS compared to those in the high radscore group. These findings indicate that the radiomics signature can serve as a useful tool in predicting the survival benefit of immunotherapy in GC.

### Immune infiltration in the high and low radscore groups

**Fig. 5:**
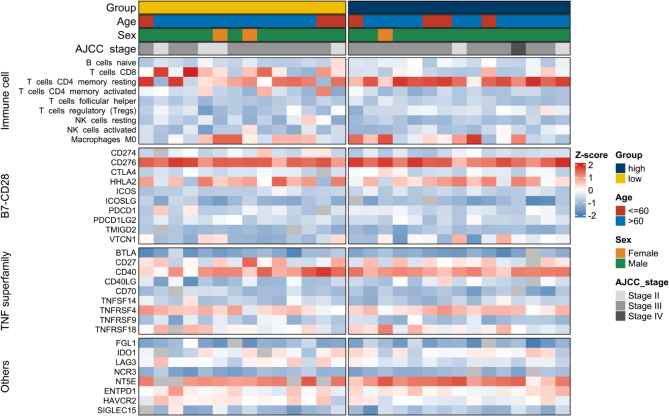
The heatmap of the clinical- and immune-related molecular landscape. From the top to the end, there are five categories, encompassing clinical characteristics, immune cells, B7-CD28, TNF superfamily, and other immune-related molecular landscapes

**Fig. 6:**
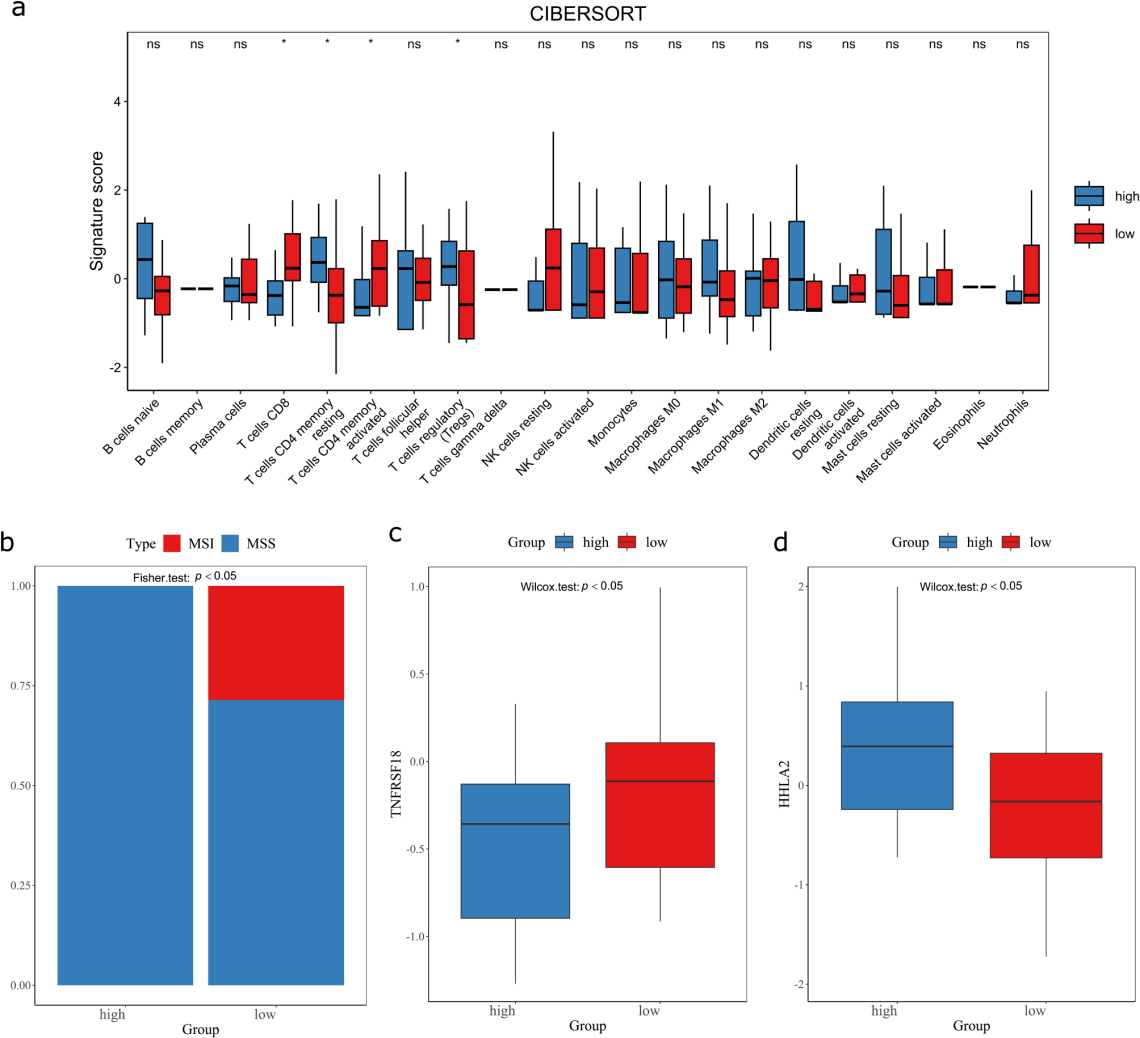
Immune cell infiltration (**a**), proportion of microsatellite instability (MSI) status (**b**), expression status of TNFRSF18 (**c**) and expression status of HHLA2 (**d**) in different radscore groups

The immune infiltration landscape of both groups is depicted in Fig. 5. The results indicated an activated immune microenvironment in the low radscore group but a potentially immunosuppressive state in the high radscore group. Specifically, the low radscore group demonstrated significantly higher CD8 + T cell levels compared to the high radscore group (Fig. 6a). Although memory resting CD4 + B cells were lower, activated CD4 + B cells were significantly higher in the low versus high radscore group (Fig. 6a). Moreover, regulatory T cells (Tregs) were increased in the high radscore group compared to the low group (Fig. 6a). Additionally, the proportion of microsatellite instability (MSI) was notably higher in the low radscore group (Fig. 6b), along with increased TNFRSF18 expression (Fig. 6c). Meanwhile, HHLA2 expression was significantly elevated in the high versus low radscore group (Fig. 6d).

## Discussion

The detection of MSI status is crucial to guide immunotherapy for GC patients, with MSI-H as a predictive biomarker [12]. We developed and validated a non-invasive radiomics signature with good performance to predict MSI-H in GC. Importantly, our investigation revealed an association between the signature and immunotherapy response and outcomes in multi-center cohorts. Additionally, findings exhibited an activated immune microenvironment in the low radscore group, while the high radscore group showed immunosuppression. This underscores the biological significance of our signature in predicting immunotherapy efficacy.

The prevalence of MSI-H in GC has been reported to vary from 5.6 to 33.3% in previous studies, possibly due to differences in detection methods. In this study, we used PCR as the gold standard for detecting MSI-H and observed an incidence rate of 22.4% in our study population. Compared to the deficient DNA mismatch repair (MMR) immunohistochemistry (IHC) method commonly used in most studies, PCR can more accurately reflect the MSI status. Furthermore, the IHC method is highly dependent on sample quality, and samples may gradually lose nucleic acids or proteins over time, leading to decreased detection accuracy [26].

Previous studies have demonstrated that certain clinical characteristics, such as age, gender, and tumor location, are significantly correlated with GC MSI [27]. A recent meta-analysis [28] found that women have a significantly higher probability of exhibiting MSI than men (OR 1.57, 95% CI: 1.31 to 1.89; *p* < 0.001), and that MSI is significantly associated with those aged 65 years and older (OR 1.58, 95% CI: 1.13 to 2.20; *p* < 0.001) and upper GC location (OR 0.38, 95% CI: 0.32 to 0.44; *p* < 0.001). In this study, MSI-H expression was notably correlated with tumor location, consistent with previous research. The pathogenesis of GC varies depending on its location, leading to distinct expressions of phenotype markers, biological behaviors, and gene expression profiles [29, 30]. This may be the biological basis for the higher expression of MSI-H in gastric antrum cancer. However, age and gender did not demonstrate significant correlations with MSI, possibly due to a limited sample size and data bias. Moreover, MSI exhibited notable correlations with Lauren classification (intestinal subtype), TNM stage, and other pathological features [31]. However, as the aim of this study was to establish a pre-treatment non-invasive prediction model, these factors were not taken into consideration.

In addition to clinical and pathological features, several studies have indicated that CT characteristics may hold value as predictors of MSI status. A retrospective study of 77 patients revealed that dMMR GC typically displays a lower stomach location, smaller tumor thickness and lymph node diameter, and fewer lymph nodes on CT imaging [32]. Another study conducted by Wu et al., which analyzed clinical and pathological information from 114 patients with colorectal cancer, showed that multi-parameter analysis derived from single-source dual-energy CT can relatively accurately distinguish between MSI and MSS in colorectal cancer [33]. Nevertheless, further large-scale studies are necessary to validate these findings.

Radiomics holds significant promise in predicting the MSI status of tumors [34–36]. While the precise mechanisms through which radiomics predicts MSI status remain incompletely elucidated, it is hypothesized that radiomics can capture tumor heterogeneity and forecast genetic alterations [15, 26]. Although previous radiomics investigations have predominantly centered on colorectal cancer, limited research has addressed predicting MSI status in GC. For instance, Zhao et al. constructed a clinical-radiomics combined model capable of predicting GC’s MSI status, yielding an AUC of 0.836 (95% CI: 0.780–0.893) in the training cohort and 0.834 (95% CI: 0.688–0.981) in the externally validated cohort [21]. Similarly, Liang et al. presented a radiomics model with AUC values of 0.823 (95% CI: 0.736–0.910) and 0.760 (95% CI: 0.663–0.858) in the training and external validation cohorts, respectively [22]. However, they did not further confirm its clinical value in immunotherapy effectiveness and prognosis, nor did they deeply investigate its biological mechanisms.

In this study, we utilized the LASSO algorithm to construct a radiomics signature associated with MSI-H expression in GC. The top-ranking features, determined by their coefficient weights, include original_glcm_MCC, original_gldm_Large Dependence Low Gray Level Emphasis, and wavelet.LHL_glszm_Large Area High Gray Level Emphasis. These features offer insights into image texture complexity (MCC), gray level distribution (Large Dependence Low Gray Level Emphasis), and gray heterogeneity (Large Area High Gray Level Emphasis). The findings indicated that MSI-H gastric cancer potentially exhibits a more intricate texture structure, decreased gray heterogeneity, and a more uniform gray level distribution. Furthermore, our results demonstrated that the signature displayed promising performance, with an AUC of 0.851 (95% CI: 0.782, 0.919) in the training cohort and 0.816 (95% CI: 0.706, 0.926) in the validation cohort. These AUC values surpassed those reported in previous studies, underscoring the potential clinical utility of our signature.

Moreover, this study extended its investigation to assess the radiomics signature’s predictive capacity for immunotherapy efficacy and prognosis. Notably, patients with a low radscore demonstrated a higher likelihood of achieving CR/PR following immunotherapy. The radiomics signature exhibited predictive AUC values of 0.734 (95% CI: 0.662, 0.806) and 0.724 (95% CI: 0.572, 0.877) for CR/PR in the ZZU and SDU cohorts, respectively. Importantly, patients identified as low radscore through the radiomics signature displayed notably extended median progression-free survival (PFS) and overall survival (OS) compared to those in the high radscore group, suggesting its potential role in anticipating the survival benefits of immunotherapy in GC. Significant parallels can be drawn from Huang et al.‘s extensive multi-cohort GC study [37], which established a CT-based radiomics score (RS) using 2272 patients. This study explored the correlation between the radiomics biomarker and the neutrophil-to-lymphocyte ratio (NLR) within the tumor immune microenvironment, including its link to prognosis and immunotherapy response in advanced GC. Huang et al. found that patients with lower RS (60.9% and 42.9%) exhibited substantially higher objective responses to anti-PD-1 immunotherapy compared to those in the higher RS group (8.1% and 14.3%). Moreover, Sun et al. integrated various solid tumor patient cohorts and developed an independently validated radiomics biomarker for tumour-infiltrating CD8 cells [38]. They demonstrated its correlation with the tumor immune phenotype and its predictive potential for clinical outcomes in patients undergoing immunotherapy. These studies collectively underscore the micro-level role of radiomics features in predicting immunotherapy outcomes through the discrimination of the tumor immune microenvironment.

Considering this, the study conducted further analysis in the TCIA cohort to investigate the MSI expression status and immune infiltration in the two subgroups. The results consistently demonstrated a significantly higher proportion of MSI in the low radscore group, providing additional evidence for the strong correlation between the radiomics signature and MSI status. Notably, the immune infiltration analysis revealed compelling findings. The low radscore group exhibited significantly elevated levels of CD8 + T cells and CD4 + B cells activated compared to the high radscore group, indicating enhanced activation and functionality of these effector immune cells. On the other hand, the high radscore group showed notably higher levels of CD4 + B cells memory resting and Tregs compared to the low radscore group, suggesting a more immunosuppressive microenvironment with increased regulatory T cell presence. Furthermore, the low radscore group displayed significantly higher TNFRSF18 expression, while the high radscore group exhibited significantly elevated levels of HHLA2. TNFRSF18 is involved in immune activation, promoting T cell proliferation and enhancing anti-tumor immune responses, while HHLA2 plays a role in immune checkpoint regulation, suppressing T cell activation and contributing to immune evasion in the tumor microenvironment. Collectively, these comprehensive results provide further insights into the immune landscape associated with the radiomics signature. The observed activated immune microenvironment in the low radscore group and the immunosuppressive state in the high radscore group contribute to a better understanding of the underlying biological significance of the radiomics signature in predicting immunotherapy outcomes.

Our study has some limitations that need to be considered. Firstly, this is a retrospective study, which may inevitably lead to some selection bias in the collected information. Secondly, in the current study, we exclusively employed the median as the grouping threshold. Depending solely on a singular threshold, like the median, for patient stratification as a biomarker might prove insufficient to accommodate the intricacies of clinical practice [39]. Thirdly, the methods for assessing MSI status differed between the cohort from Center 1 and the TCIA cohort. In addition, despite being a multicohort study, this research had a relatively small sample size and relied on publicly available databases. Future prospective multi-center studies with larger sample sizes are warranted to further validate the findings of this study.

In conclusion, our study developed a novel radiomics signature for predicting MSI-H expression in GC patients, guiding immunotherapy and predicting clinical outcomes. The radiomics signature also unveiled distinct immune profiles between low and high radscore groups, underscoring their clinical relevance. These findings emphasize the potential of radiomics analysis as a non-invasive tool for tumor characterization and personalized treatment selection in GC, warranting further validation in diverse populations and clinical settings to establish its clinical implications.

### Electronic supplementary material

Below is the link to the electronic supplementary material.

Supplementary Material 1

Supplementary Material 2

Supplementary Material 3

## Data Availability

The datasets used and/or analysed during the current study available from the corresponding author on reasonable request.

## Abbreviations

MSI: Microsatellite instability
GC: Gastric cancer
TCIA: The Cancer Imaging Archive
LASSO: The Least Absolute Shrinkage and Selection Operator
AUC: Receiver operating characteristic curve
PFS: Progression-free survival
OS: Overall survival
PCR: Polymerase chain reaction
CI: Confidence interval
CR: Complete response
PR: Partial response
SD: Stable disease
PD: Progressive disease
