# Supplementary material for: A radiomics signature derived from CT imaging to predict MSI status and immunotherapy outcomes in gastric cancer: a multi-cohort study
Source: BMC Cancer. 2024 Apr 1;24:404. doi: 10.1186/s12885-024-12174-0 (PMC10985890; doi:10.1186/s12885-024-12174-0)
Supplement: Supplementary file 1 — Supplementary Material 1 [file 12885_2024_12174_MOESM1_ESM.doc]

**Supplementary A: Inclusion and exclusion criteria**

**The primary cohort**

The inclusion criteria: (1) histologically confirmed gastric adenocarcinoma; (2) with confirmed MSI status by PCR detection; (3) underwent CECT within two weeks prior to anti-tumor treatment.

The exclusion criteria: (1)poor image quality; (2) poor gastric filling; (3) history of anti-tumor treatment before CECT.

**The immunotherapy cohorts**

The inclusion criteria: (1) histologically confirmed gastric adenocarcinoma; (2) diagnosed with metastatic GC based on pre-treatment CECT examination; (3) received at least three cycles of PD-1/PD-L1 inhibitor; and (4) underwent standard baseline CECT before treatment.

The exclusion criteria: (1) lack measurable lesions that met the Response Evaluation Criteria in Solid Tumors (RECIST 1.1); (2) lack evaluation CECT scans after three cycles of anti-PD-1/PD-L1 treatment; (3) lack follow-up.

**The TCIA cohort**

The inclusion criteria: (1) histologically confirmed gastric adenocarcinoma; (2) with available CECT images and mRNA sequencing data.

The exclusion criteria: (1)poor image quality; (2) poor gastric filling; (3) prior anti-tumor treatment or gastric tube placement to CECT; (4) lack transcriptome annotation information.

**Supplementary B: Immunotherapy regimens used in the immunotherapy cohorts**

All gastric cancer patients of the immunotherapy cohorts receive an immunotherapy regimen consisting of PD-1/PD-L1 inhibitors in combination with first-line chemotherapy. The chemotherapy regimen mainly includes oxaliplatin/cisplatin plus fluorouracil/capecitabine/paclitaxel. The PD-1/PD-L1 inhibitors and their administration methods are as follows: (1) Pembrolizumab Injection (Merck & Co., Kenilworth, USA), administered intravenously at a dosage of 200 mg every 3 weeks; (2) Sintilimab Injection (Innovent Biologics, Suzhou, China), administered intravenously at a dosage of 200 mg every 3 weeks; (3) Nivolumab Injection (Bristol-Myers Squibb Company, New York, USA), administered intravenously at a dosage of 3 mg/kg every 2 weeks.

**Supplementary C: CT Acquisition**

To prepare for the abdominal CECT examination, patients were instructed to fast for 12 hours to empty their gastrointestinal tract. Additionally, an intramuscular injection of 20 mg scopolamine was administered 20 minutes prior to imaging to reduce gastrointestinal peristalsis. To ensure optimal gastric filling, patients were also instructed to drink 800-1000mL of warm water approximately 5 minutes before the examination.

Two different CT scanners, Revolution CT (GE Healthcare, United States) and Aquilion ONE (Otawara, Japan), were used to perform the abdominal CECT examinations. For Revolution CT, the image acquisition parameters were as follows: tube voltage of 120 kV, automatic tube current of 240-450 mA, pitch of 0.992:1, rotation time of 0.5 s, detector width of 80 mm, reconstruction algorithm of STAND, scan slice thickness of 5 mm, and reconstructed section thickness of 0.625 mm. For Aquilion ONE, the image acquisition parameters were as follows: tube voltage of 120 kV, tube current of 350 mA, rotation time of 0.5 s, scan slice thickness of 5 mm, and reconstructed section thickness of 2 mm.

After an unenhanced CT scan was performed, an intravenous contrast agent (Ultravist 370, Bayer Schering Pharma, Germany) was injected via antecubital venous access at a rate of 3.0 mL/s, for a total iodine dose of 1.5 mL/kg, followed by a 20-mL saline flush. Arterial phase (AP) and venous phase (VP) CT images were obtained after a post-injection delay of 30 and 70 seconds, respectively.
