# Supplementary material for: A radiomics signature derived from CT imaging to predict MSI status and immunotherapy outcomes in gastric cancer: a multi-cohort study
Source: BMC Cancer. 2024 Apr 1;24:404. doi: 10.1186/s12885-024-12174-0 (PMC10985890; doi:10.1186/s12885-024-12174-0)
Supplement: Supplementary file 2 — Supplementary Material 2 [file 12885_2024_12174_MOESM2_ESM.doc]

**Table E1: Characteristics of the TCIA cohort**

|  | TCIA cohort (n = 29) | | *p* value |
| --- | --- | --- | --- |
| Characteristic | Low radscore (n = 14) | High radscore (n = 15) |  |
| Age (years) |  |  | >0.999 |
| ≤60 | 3 (21.43) | 4 (26.67) |  |
| >60 | 11 (78.57) | 11 (73.33) |  |
| Sex |  |  | 0.598 |
| Male | 12 (85.71) | 14 (93.33) |  |
| Female | 2 (14.29) | 1 (6.67) |  |
| AJCC stage |  |  | 0.403 |
| II | 4 (28.57) | 2 (13.33) |  |
| III | 10 (71.43) | 12 (80.00) |  |
| IV | 0 (0) | 1 (6.67) |  |

Note: Except where indicated, data are number (%) of patients.

**Table E2: Coefficients of each feature in the radiomics signature**

| Features | Coefficients |
| --- | --- |
| original_glszm_SmallAreaLowGrayLevelEmphasis | -0.05980119 |
| wavelet.LHH_firstorder_Skewness | -0.026505875 |
| original_ngtdm_Busyness | -0.075894979 |
| original_glrlm_RunEntropy | 0.101316788 |
| wavelet.LLH_glcm_Correlation | 0.0240378 |
| wavelet.LHH_firstorder_Median | 0.04509294 |
| original_glcm_MCC | 0.328493539 |
| wavelet.LHL_glszm_LargeAreaHighGrayLevelEmphasis | -0.128687026 |
| original_gldm_LargeDependenceLowGrayLevelEmphasis | -0.358872649 |
| Intercept | 1.301708388 |

**Table E3: Results of Delong test for ROC comparison**

| Model | Cohort |  | Model | | |
| --- | --- | --- | --- | --- | --- |
|  |  |  | Clinical | Radiomics signature | Combined model |
| Clinical | Training |  | / | *p* = 0.005 | *p* < 0.001 |
|  | validation |  | / | *p* = 0.041 | *p* = 0.504 |
| Radiomics signature | Training |  | *p* = 0.005 | / | *p* = 0.196 |
|  | validation |  | *p* = 0.041 | / | *p* = 0.711 |
| Combined model | Training |  | *p* < 0.001 | *p* = 0.196 | / |
|  | validation |  | *p* = 0.504 | *p* = 0.711 | / |

Data are *p* values calculated by Delong test for ROC comparison between different models in the training and validation cohorts. ROC, receiver operating characteristic curve.
