# Supplementary material for: A radiomics signature derived from CT imaging to predict MSI status and immunotherapy outcomes in gastric cancer: a multi-cohort study
Source: BMC Cancer. 2024 Apr 1;24:404. doi: 10.1186/s12885-024-12174-0 (PMC10985890; doi:10.1186/s12885-024-12174-0)
Supplement: Supplementary file 3 — Supplementary Material 3 [file 12885_2024_12174_MOESM3_ESM.doc]

**The logistic regression formulas for the three models:**

The clinical model: logit(*p*) = -2.079 + 1.609×Antrum + 0.345×Body + 0.693×Whole

The radiomics model: logit(*p*) = 1.791 - 2.573×radiomics signature

The combined model: logit(*p*) = 0.991 + 1.280×Antrum + 0.419×Body - 1.115×Whole - 2.480×radiomics signature

Note: *p* represents the probability of MSI-H, Antrum, Body and Whole take values of 0 or 1, and the radiomics signature can be calculated using the coefficients presented in Supplementary Table E2.

**The validation results of the radiomics model using nested 5-fold cross-validation, repeated 5 times**

| **Folds** | **Repeat times** | **Area under the ROC curve** |
| --- | --- | --- |
| 1 | 1 | 0.819 |
| 2 | 1 | 0.724 |
| 3 | 1 | 0.753 |
| 4 | 1 | 0.842 |
| 5 | 1 | 0.857 |
| 1 | 2 | 0.885 |
| 2 | 2 | 0.692 |
| 3 | 2 | 0.879 |
| 4 | 2 | 0.882 |
| 5 | 2 | 0.824 |
| 1 | 3 | 0.774 |
| 2 | 3 | 0.756 |
| 3 | 3 | 0.807 |
| 4 | 3 | 0.735 |
| 5 | 3 | 0.842 |
| 1 | 4 | 0.649 |
| 2 | 4 | 0.951 |
| 3 | 4 | 0.785 |
| 4 | 4 | 0.767 |
| 5 | 4 | 0.885 |
| 1 | 5 | 0.828 |
| 2 | 5 | 0.878 |
| 3 | 5 | 0.767 |
| 4 | 5 | 0.785 |
| 5 | 5 | 0.719 |
